# King’s College Hospital

**Published:** 1852-11

**Authors:** 


King's College Hospital. *Consulting Physicians*—Drs. Watson and Ferguson. *Physicians*—Drs. Budd and Todd. *Physician Accoucheur*—Dr. A. Farre. *Physician to Out-Patients*—Dr. Guy. *Assistant Physician*—Dr. G. Johnson. *Surgeons*—Messrs. Fergusson and Partridge. *Assistant Surgeons*—Messrs. Bowman and Lee. *Surgeon Dentist*—Mr. Cartwright.

King's College, Medical Department.—Anatomy, Descriptive and Surgical, Mr. Partridge. Demonstrations, Messrs. Lee, Salter, and Wood. Physiology, General and Morbid Anatomy, Drs. Todd and Bowman. Chemistry, Dr. W. A. Miller. Medicine, Dr. Budd. Surgery, Mr. Fergusson. *Summer Session.*—Midwifery, Dr. A. Farre. Materia Medica and Therapeutics, Dr. Royle. Botany, Mr. Forbes. Forensic Medicine, Dr. Guy. Practical Chemistry, Mr. J. E. Bowman, Comparative Anatomy, Mr. R. Jones.

